# Foreign Body Granulomas Reaction Related to Collagen Stimulatory Cosmetic Fillers: A Systematic Review

**DOI:** 10.1111/jocd.70459

**Published:** 2025-10-24

**Authors:** Hang Wang, Dongmei Wu, Chihchieh Lo, Shuo Liu, Qiang Ji, Al‐Attab Reem, He Qiu

**Affiliations:** ^1^ Department of Cosmetic and Plastic Surgery, West China Hospital of Stomatology Sichuan University Chengdu Sichuan China; ^2^ Department of Cosmetic and Plastic Surgery, West China Second University Hospital Sichuan University Chengdu Sichuan China; ^3^ National Engineering Research Center for Biomaterials Sichuan University Chengdu Sichuan China; ^4^ Department of Cosmetic and Plastic Surgery West China School of Public Health and West China Fourth Hospital, Sichuan University Chengdu Sichuan China

**Keywords:** collagen stimulators, foreign body granuloma, foreign body reactions, treatment recommendations

## Abstract

**Background:**

Collagen‐stimulating filler has boomed as a new welcoming option in aesthetic treatments by activating the synthesis of endogenous collagen. However, hyperactive or inappropriate foreign‐body stimulatory reactions may trigger intractable foreign body granulomas (FBGs). A comprehensive understanding of the FBGs associated with the augmenting effect of collagen biostimulants can help clinicians perform treatments in a safer and more effective way.

**Methods:**

A literature search was conducted in Web of Science, PubMed, Embase, and Scopus to identify relevant clinical reports about the FBGs induced by six types of commercially available collagen stimulators up to August 2024.

**Results:**

A total of 40 studies involving 117 patients were included. All but eight cases were women, and the mean age was 52.63 years (range 29–78 years). The most reported cases of FBGs were associated with PMMA (35.04%), followed by PLLA (30.77%), CaHA (27.35%), PCL (4.27%), and dextran‐based filler (2.56%). The time until initial detection of FBGs reaction ranged from 1 week to 15 years (mean 20.18 months). Nodules were the most frequent complication (82.91%), followed by swelling (9.40%) and lumps (3.42%). The perioral region was the reported high‐risk region for FBGs. Intralesional injection of steroids was performed most frequently (21.37%), but symptoms were often resolved through surgical removal.

**Conclusions:**

All included collagen biostimulators can induce FBGs reactions, with the nondegradable fillers having a higher reported incidence and a longer latency period. Timely recognition, diagnosis, and appropriate management of FBGs behind tissue augmentation with collagen stimulator filler are necessary for clinicians to optimize efficacy and minimize adverse events.

**Level of Evidence:**

V.

With the increased demand for facial rejuvenation and a shift toward a long‐term restoration effect, collagen stimulatory fillers for facial augmentation have dramatically gained popularity as an alternative to traditional volume‐based procedures, such as the injection of hyaluronic acid (HA) derivatives. These approved synthetic fillers are prominent representatives of collagen stimulants, such as polymethyl methacrylate (PMMA), polyvinyl alcohol (PVOH), polylactic acid (PLLA), polycaprolactone (PCL), calcium hydroxyapatite (CaHA), and dextran containing fillers (Table S1). As an injectable dermal fillers, they were formulated into microspheres or particles and suspended in different kinds of carrier medium. These biostimulatory fillers provide initial volume replacement as well as the late additional neocollagenesis for sustained facial volumization and skin quality. Several lines of evidence suggest that the biological mechanisms behind the augmenting effect most likely depend on the subclinical foreign body tissue reaction of the filler, mediated by moderate inflammatory cell infiltration, foreign body cell recognition, fibroblast growth, material encapsulation, and the consequent collagen deposition. Notably, these synthetic particulate materials, as potential foreign bodies, may also clinically cause a series of rare foreign body reactions (FBRs) during the intended process of promoting collagen regeneration, such as hypopigmentation, lumps, nodular masses, hypertrophic scars, localized swelling, fibrosis, severe skin necrosis, chronic infections, or even serious autoimmune diseases [1, 2]. These semipermanent or permanent fillers were more likely to be associated with lumps and nodules than other complications, generally occurring late (> 1 month) [3]. Furthermore, certain material characteristics may play a vital role in bringing about microsphere‐containing fillers more prone to these FBRs than others. Foreign body granuloma (FBGs) is the main manifestation of chronic FBRs and is classified as a grade 4 FBR. The classification of FBRs described by Duranti et al. shows the classic histopathological features of foreign body granulomas (FBGs), that is, granulomas with encapsulated implants and clear foreign body reactions [4]. Lemperle G's comprehensive review concluded that the FBGs of particulate injectables typically present as sclerosing granulomas, and the possibility of a blend of cystic or edematous granulomas may also exist [5].

Studies of the FBRs of particulate fillers such as these suggested that FBGs are a rare, intractable, and recurrent presentation that requires immediate attention [6, 7, 8, 9]. A particularly challenging problem is that permanent fillers often lack effective solvents, leading to complications that are typically persistent. Data from several studies suggested that the incidence of complications in nonabsorbable dermal fillers was nearly 10 times higher than that of absorbable fillers [10, 11, 12]. In addition, permanent products, like PMMA and PVOH fillers, are more likely to be the origin of unaesthetic, recurrent, and invalidating FBGs that are often very long‐lasting and resistant to various local treatments [13]. However, many of biomolecular and clinical studies up to now have been extensively focusing on the augmenting effect of stimulatory filler. There remains a paucity of evidence on the long‐term safety, especially the nonbiodegradable stimulatory filler. Herein, we present the first systematic review and general discussion regarding to the status and management of FBGs related to the collagen stimulants to better guide practitioners in facilitating safer clinical procedures.

## 1 Literature Review

**FIGURE 1:**
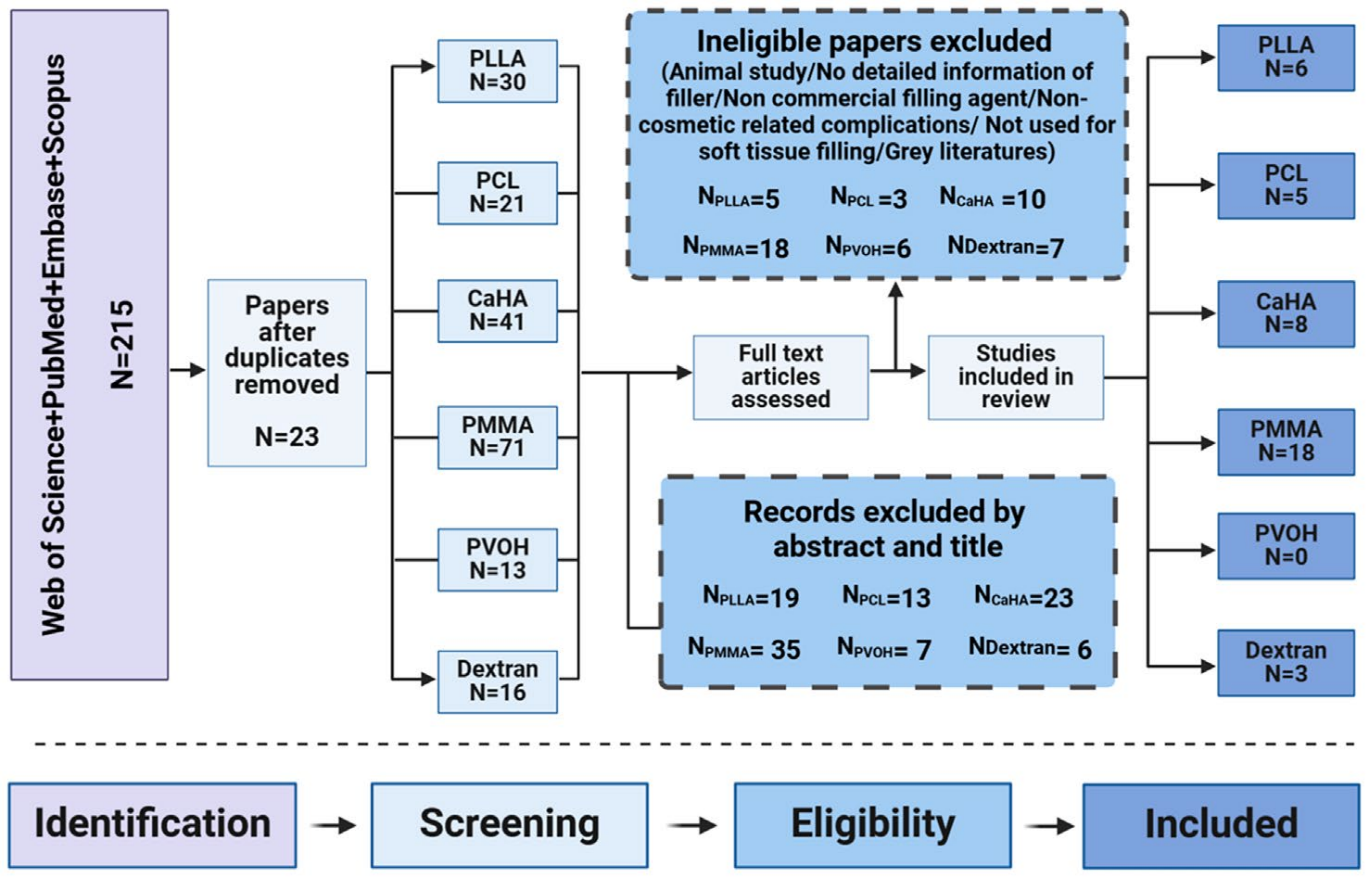
The flow diagram of the literature screening and study selection process.

The search strategy for this study was guided by the Population, Intervention, Comparison, Outcome (PICO) framework [14]. The detailed Medical Subject Headings (MeSH) terms retrieved and search strategies for this research were shown in Appendix S1. The fillers retrieved above were as shown in Table S1. An extensive literature search was performed by three reviewers (H.Q., H.W., and S.L.) via Web of Science, PubMed, Embase, and Scopus in August 2024 to identify all reports pertaining to FBGs of the PCL, PLLA, CaHA, PVOH, PMMA, and dextran‐based regenerative biostimulators after cosmetic procedures. FBGs reaction induced by fillers was mainly diagnosed by clinical manifestation and pathologic examination. Two investigators (C.Lo. and Q.J.) independently conducted initial screening on abstracts and full‐text reviews of relevant studies according to the predetermined criteria (Figure 1). Any disagreements or ambiguities with the search results and data analysis were adjudicated by discussion with the senior author (DM.W.).

## 2 Results

A total of 215 articles were retrieved with the prespecified search criteria. Following the removal of duplicates, 192 studies were screened, and 89 articles underwent full‐text assessment (Figure 1). Forty studies were finally included in this systematic review. The patient clinical characteristics, types of treatments implemented, and prognosis of the included studies are detailedly presented in Table S2.

### 2.1 Clinical Characteristics of the Included Studies

**FIGURE 2:**
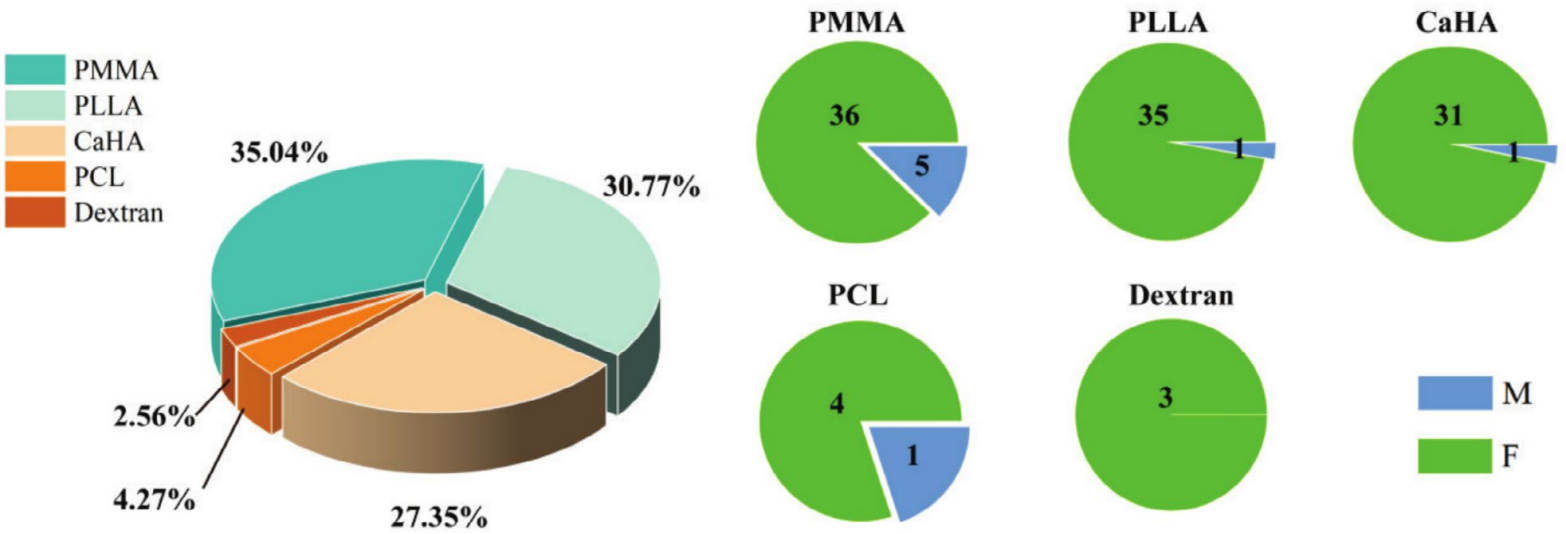
Number of patients with FBGs due to various microspheres‐containing dermal fillers (F: female; M: male).

Thirty‐nine case reports and one retrospective study describing a total of 117 patients who suffered from FBGs after receiving injections met final inclusion criteria. All 117 patients, aged 29–78 years (median, 52.63 years), were 109 women (93.16%) and 8 men (6.84%). As seen in Figure 2, PMMA (35.04%) has been reported to cause the most granulomas reactions, followed by PLLA (30.77%) and CaHA (27.35%). This result was closely related to the properties of the materials, such as immunogenicity and degradability. Six different types of dermal fillers and nine products were reported in these patients, with Artecoll (Rofil Medical, Breda, Netherlands) (*n* = 35/29.91%) and Radiesse (Merz Pharmaceuticals, Frankfurt, Germany) (*n* = 32/27.35%) being the most reported. Newfill (Sanofi Aventis, Paris, France) was reported in 20 patients (17.09%) and other fillers were also described, such as Sculptra (also known as New‐Fill; Sanofi Aventis, Paris, France), ELLANSÉ (Aqtis Medical, Utrecht, Netherlands), Matridex (BioPolymer, Samedan, Switzerland), Bioplasty (Silikon Industria, Rio de Janeiro, Brazil), and Metacrill (Nutricell, Campinas, Brazil).

**FIGURE 3:**
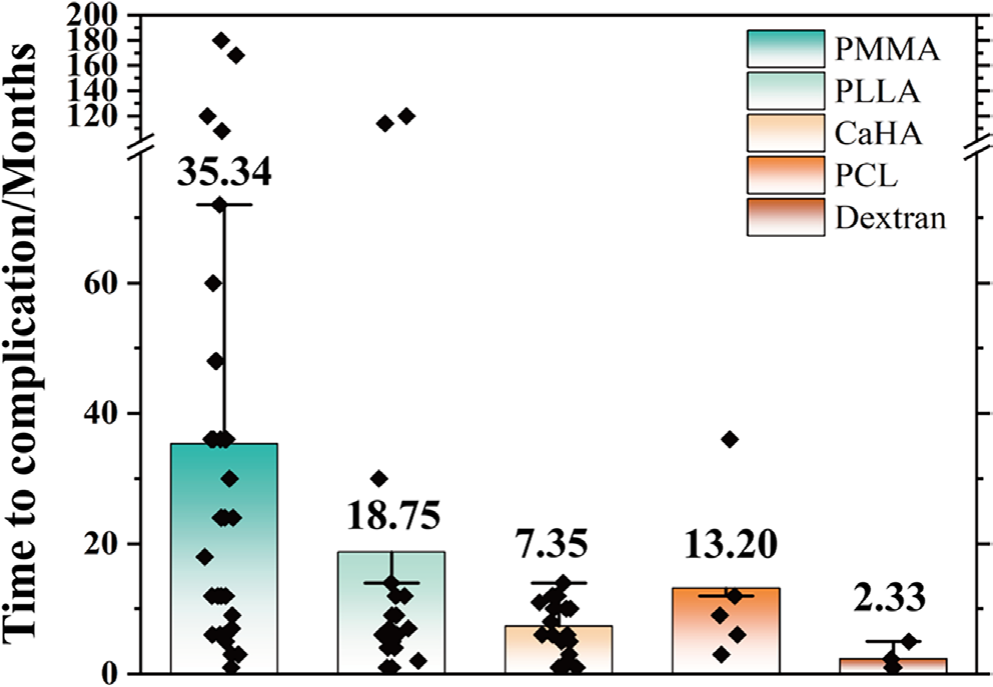
Months between filler injection and FBG's first development.

The time elapsed from injection to initial detection of the lesion ranged from 1 week to 15 years, with a median of 20.18 months. The time until complications varied by filler type: the median time was 18.75 months for PLLA, 13.20 months for PCL, 7.35 months for CaHA, 35.34 months for PMMA, and 2.33 months for dextran microspheres (Figure 3). A longitudinal study of the human histology and persistence of dextran microspheres by Lemperle G reported that early redness and swelling after implantation can occur within 10 days and may persist for up to 3 months [53]. This was consistent with the reported window period (range: 1–5 months) for FBRs of dextran microspheres and is also one of the reasons for the earliest occurrence of granuloma formation in these six fillers. As an inert implant, FBGs associated with PMMA often appeared later, sometimes as long as 15 years after initial injection, and could persist the longest.

### 2.2 Foreign Body Granulomas Reaction Presentation

**FIGURE 4:**
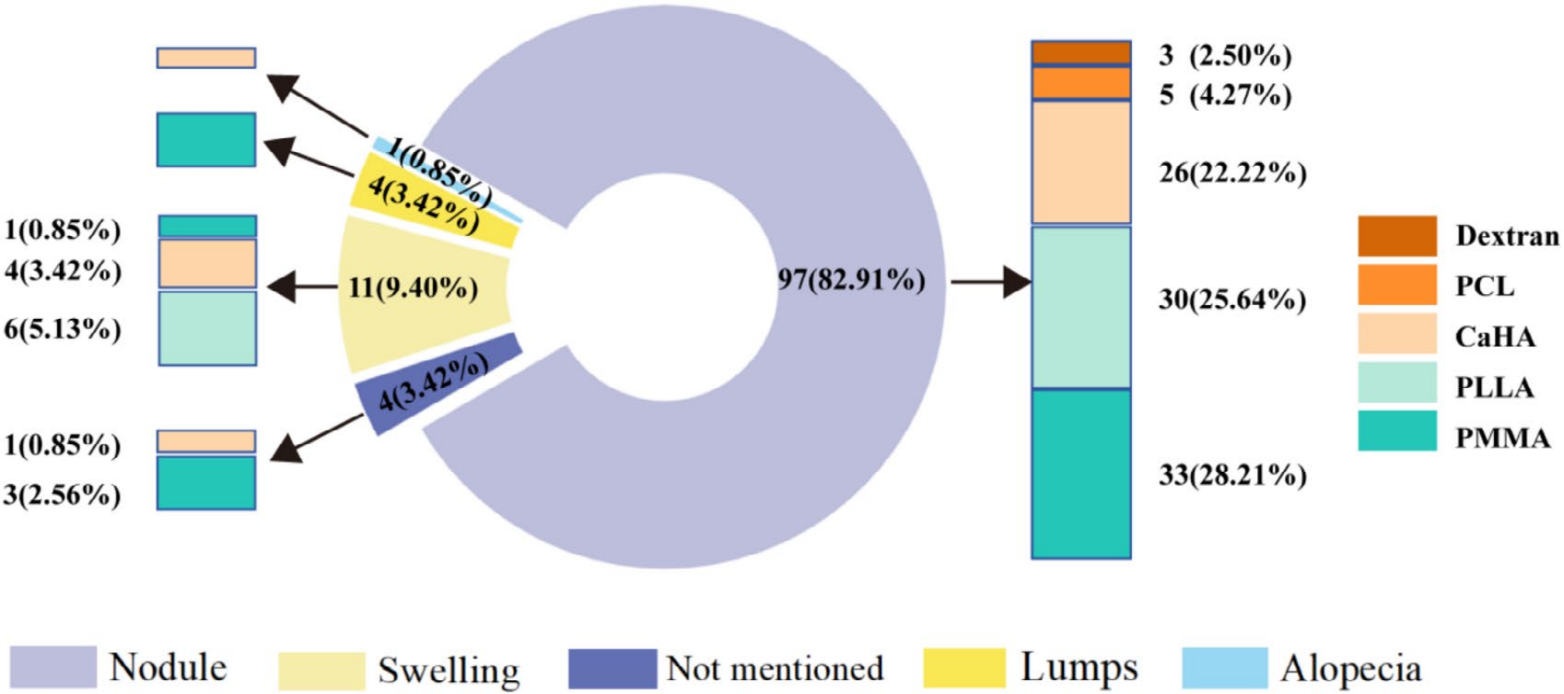
The clinical manifestations and proportions of granuloma reactions for each filler.

**FIGURE 5:**
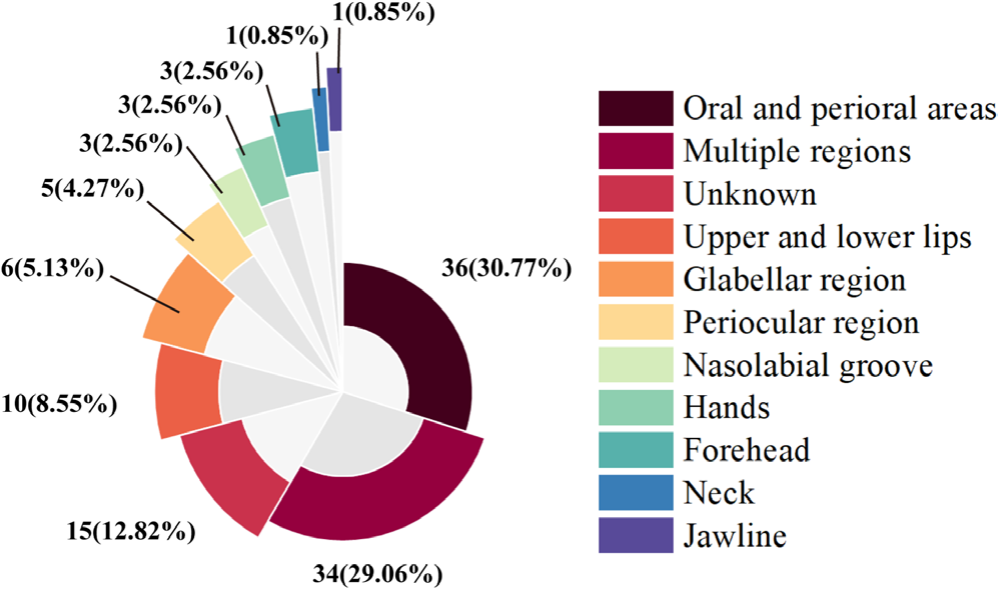
The reported sites and proportions of granuloma reactions' occurrence.

The granulomatous lesions at presentation appeared from lumps or tumefaction to the most common nodules, occasionally with migration occurrence or necrosis manifestations (Figure 4). Among the affected regions, the oral and perioral areas were the most common anatomical locations of granulomas, accounting for 30.77% of cases. This includes the labial mucosa, labial vestibule, and mental area. Other notable sites represented the common affected anatomical locations of granulomas. The lips represent 8.55% of cases, the glabellar area was 5.13%, and the periocular area was 4.27% (Figure 5). Among the multiple lesion sites, in addition to the conventional facial injection areas, other parts of the body were also involved, such as the buttocks and thighs. Occasional reports also mention the hands, jawline, and neck.

### 2.3 Treatments Implemented for Foreign Body Granulomas Reaction

**FIGURE 6:**
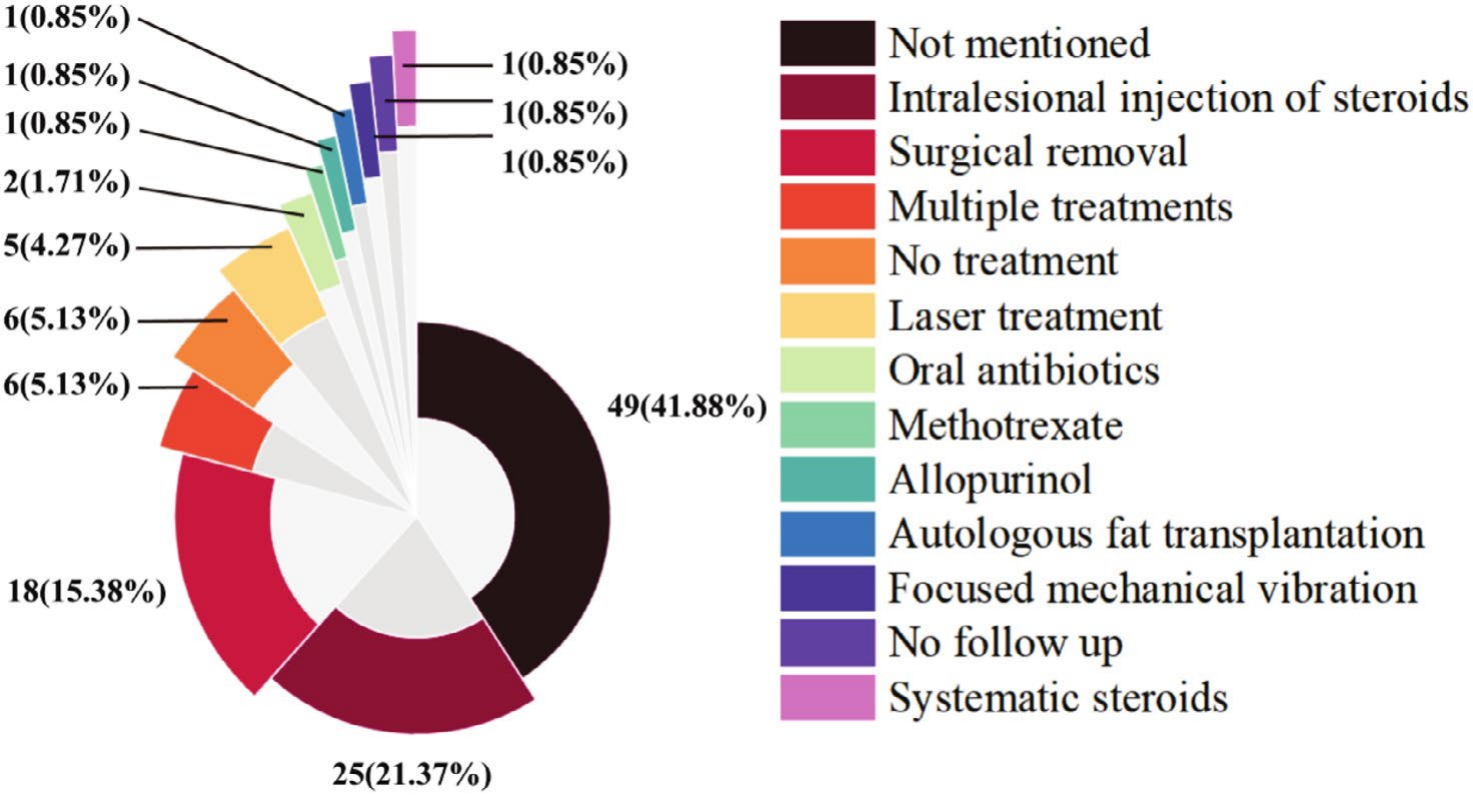
The treatment modalities of the included cases.

As is shown in Figure 6, the majority of the cases were managed by intralesional injections of steroids (25/21.37%), followed by surgical removal of lesions (18/15.38%), laser treatment (5/4.27%), and antibiotics (2/1.71%). Furthermore, allopurinol, methotrexate, and fluorouracil were also reported in other cases. However, the treatment protocol was not mentioned in 48 cases, 1 case was lost to follow‐up, and 6 cases did not receive any intervention.

### 2.4 Treatment Outcomes

**FIGURE 7:**
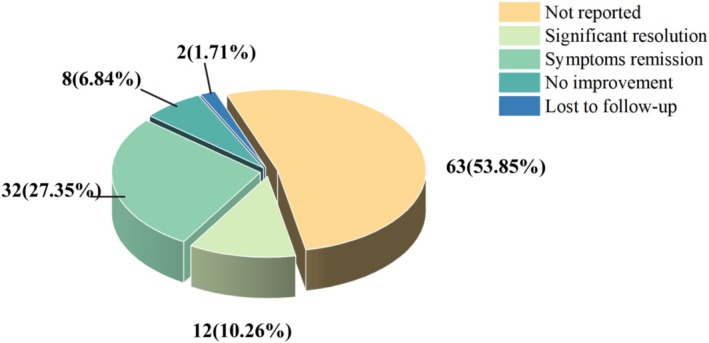
The treatment prognosis of the included cases.

Figure 7 illustrated that there was remission of the nodule (27.35%) or complete resolution (10.26%). Eight cases reported persistent lesions with no significant improvement in lesion size or symptoms. A total of 65 cases were lost to follow‐up or did not report on the outcome. It is worth noting that most refractory cases are mainly resolved through surgical excision.

## 3 Discussions

Microsphere‐based tissue stimulators were used more as a sculpting and regenerative agent rather than just a volumizing filler. These fillers are considered relatively safe, with most adverse reactions occurring either immediately or shortly after injection. Noteworthy, the long‐term or delayed‐onset adverse effects caused by any implanted medical device are often considered FBRs, which tend to occur several months to years after initial injection and usually form characteristic FBGs microscopically. Ideally, the limited FBRs around the injected material are the body's natural response to the implant. That is often presented as a monolayer of macrophages or fibrocytes surrounding microspheres and enveloped by a zone of new fibrous tissue, which is the true histologic basis of the volume restoration and not a fibrosis reaction. However, variable degrees of fibrosis with an accumulation of product were often noted in the manifestation of granulomatous FBR, especially induced by nonbiodegradable microsphere filling agents. This accumulation of tissue regeneration differs in that it is primarily due to the host tissue's excessive reaction to small amounts of the product [54]. As Werschler put it, the granulomatous reaction was not identical to the clinical biostimulator‐induced nodules, especially in the primary mechanism of action, histopathologic response, and even treatment approaches [55]. It can be seen in Table S1 that the main histological appearance of FBGs associated with collagen biostimulators varied from fillers to fillers, mainly depending on the chemical nature of the microspheres, their surface structure, and the presence of carriers. Knowing the histological characterization of FBGs is important for our increased understanding and distinguishing of different injected substances.

It has previously been observed that microsphere size was among the most important factors for FBRs. Most of these particle‐containing fillers are presented with a diameter of 30–50 μm to resist macrophage phagocytosis, but they still cannot completely avoid a series of potential FBRs, not to mention the potential impact of smaller particle size, irregular shape, rough surface, inappropriate content, and surface charge. As summarized in our review, the FBGs caused by the second‐generation PMMA filler (Artecoll), containing a higher volume of particles (< 20 μm in diameter), are significantly higher than those reported for Artefill. Another PMMA‐based product, Metacrill, may also suffer from late‐onset granulomas due to a wide microsphere size distribution (1–80 μm). Similarly, FBRs in PLLA fillers mainly occurred in the Sculptra product, which has more uniform particle sizes than those of Newfill. As would be expected, implantation with irregular surface microspheres (Bioplastique (Bioplasty, St. Paul, Minnesota), Dermalive (Dermatech, Paris, France)) is prone to have a higher probability of inducing FBRs compared to smoothly surfaced microspheres [54]. Notably, the reported cases of FBGs to PMMA (*n* = 41/35.04%) in our study are more than the cases of HA (*n* = 41/28.80%, reported in 2023). Nondegradable filler is often considered as latent “living implants” to exert long‐term stimulants for connective tissue formation. This also accords with our observations, which showed that the incidence rate of granuloma formation was higher for nondegradable fillers compared to resorbable fillers in our study. As reported, the occurrence of granuloma from PMMA can happen as long as 15 years after postinjection.

Cellulose is a common carrier for injectable microsphere materials, such as PVOH, PLLA, PCL, and CaHA. CaHA and PLLA can also use cross‐linked hyaluronic acid (HA) as an excipient. PMMA‐based injectable products usually use bovine collagen as the carrier. To some extent, the source, degradation, and by‐products of the carrier material can also induce a host response. The bovine collagen carrier in PMMA fillers may trigger allergic reactions, which could also be a potential risk factor for causing FBRs. Some authors also suggested that, except for the type of carrier itself, the biological structure of the excipient (viscosity, morphology, cohesiveness, hardness, hydrophilia, surface charge, and roughness) or processing techniques (cross‐linking form) may also be other factors influencing the outcomes of fillers [56]. Therefore, some inert and asymptomatic complications arising from the above inherent material factor of fillers tend to lean more toward noninflammatory, nonspecific immune responses. Although manufacturers often claim that these fillers have good tolerance, are nonimmunogenic, or that specific immune complications are very rare, we cannot ignore the possibility of such reactions.

Further findings revealed that the occurrence of FBGs was well influenced by the site and location of injection. As concluded by Daley et al., the migration of the particles appears to be linked to the formation of ectopic granuloma lesions, especially in areas with frequent facial movements, such as the perioral area, eyelids, lips, neck, and cheeks [15, 57].

Given the off‐label use of these fillers, the locations where FBG occurs in clinical practice are more variable than the lesion sites reported in existing literature. Evidence indicates that the dermis exhibits heightened immune reactivity, leading to an increased propensity for granuloma formation following intradermal injections compared to subcutaneous or dermal‐subcutaneous junction administration. These lyophilized powder products, such as fillers containing PLLA and CaHA, often require on‐site preparation before injection. The carrier matrix and microspheres typically need to be blended or dispersed in sterile distilled water or saline to obtain a homogeneous mixture. Therefore, too superficial an injection, a high concentration of microspheres within the filler, a lack of massage, an excessive injection dose, insufficient intervals, incorrect product preparation, or hydration time may also lead to the focal aggregation of microspheres and then the induced nonimmune reactions, such as the asymptomatic nodule [44].

Our review presented the finding that the nodules in reported complications were mostly characterized as inflammatory granulomas. Many studies examined that bacteria inoculation during injection through contiguous direct extension or hematological spread may form biofilms. This can resist bodies and external degradation measures on the filler materials [58, 59, 60]. With polysaccharide‐rich heterogeneous structures embedding various microorganisms, these biofilms can evade the immune system and effectively inhibit macrophage phagocytosis. Studies have shown that these biofilms often act as bacterial antibiotic resistance and tolerance [61, 62, 63]. This may be one of the reliable explanations for the delayed foreign body reactions, such as persistent nodule granulomas. Additionally, Lemperle et al. have concluded that there may be a possible correlation between incidents provoking alterations in immune dynamics, like facial trauma, face‐lift operation, systemic infections, flu‐like syndrome, and herpes labialis, and FBGs before their clinical onset [5]. Some researchers stated certain medications, such as interferon‐α and ribavirin, that may induce skin sarcoidosis can act as a triggering cause for the occurrence of granulomatous reactions of the preexisting filler [38]. Exceptionally sensitive conditions or suffering from autoimmune diseases may also be an increased predisposing factor in FBRs causation. It seems possible that these hosts’ immune statuses or the interaction with immunostimulatory medications may stimulate the memory of macrophages and prompt a new attack, especially in cases in which filler is residual or a second injection [37, 44]. In this case, there is a probability that FBGs have cyclic periods of activation and remission in clinic. Collectively, further studies need to be conducted to elucidate the mechanisms that cause late‐onset granuloma formation.

The timing and progress of management actions are fundamental to the outcomes of resolve, remission, and exacerbation. These products that require reconstitution with excipients may cause early to immediate‐stage nodules after injection, which could be the result of particle accumulation rather than a granulomatous reaction. Surveys such as that conducted by McCarthy have shown that focused mechanical vibration relying on the combination of aqueous diluent and microneedling vibration seemed to be a promising strategy for the rapid reversal of noninflammatory nodules resulting from CaHA‐based injections [45]. For such nodules, manual dispersion of particles like local massage, mechanical vibration, or dilute saline can be considered as treatment options. There were also studies reporting that for such nodules, symptoms may improve even without any intervention by the spontaneous biodegradation of the filler [18].

As for some later‐stage ones, being managed with intralesional steroid, 5‐fluorouracil, methotrexate, bleomycin, and prednisone injections, or combined with oral agents (minocycline, allopurinol, colchicine, cyclosporine, corticosteroids, methotrexate, and doxycycline), and energy‐based devices (LED, erbium laser, intense pulsed light, fractionated laser, CO_2_ laser, and microfocused ultrasound) have been typically suggested as the symptomatic treatments. However, researchers found some of the above treatments were not entirely effective, which may stem from the classifications and types of granulomatous inflammation [18, 64]. For long‐standing nodules with fibrous encapsulation, appropriate tissue dispersing agents such as collagenase or hyaluronidase can be selected based on the type of excipients used. Emer has shown that FBRs involving hyaluronic acid carriers for adjustable fillers could be resolved or remit through hyaluronidase intervention [65]. Given the anti‐edematous and anti‐inflammatory properties of hyaluronidase, numerous studies also suggested that hyaluronidase can be used as an emergency treatment for complications associated with non‐HA‐containing fillers such as PLLA and PCL fillers [66, 67]. Another important finding was that sodium thiosulfate has the potential to disintegrate CaHA and showed the potential reversibility of CaHA‐based filler associated complications [68, 69]. For the etiological treatment, data about the promising antidote of other collagen biostimulators has not yet been investigated at present.

As Park pointed out, surgical intervention is usually the final useful option for dealing with some intractable or permanent granulomas [70]. Due to the infiltrative nature of granulomas, it is difficult to completely remove lesions through surgery. This is also the reason why postoperative discomfort persists or lesions recur in some cases. Despite the availability of various treatment methods, resolving the FBGs remains challenging. As illustrated above, there is still no single and definitive efficacious treatment, often requiring combined interventions with caution.

### 3.1 Study Limitations

It could be found that the available evidence regarding granulomatous reactions associated with collagen stimulators was mainly based on case series, and only one study is a multicenter retrospective study. Not all cases provided histological information for diagnosing FBGs. Few cases included in the review of FBGs are based only based on a comprehensive evaluation of the patient's medical history, clinical presentation, and other auxiliary diagnostic methods. Another significant drawback is the lack of standardized, objective, and comprehensive definitions of the FBGs, which may lead to retrieval imperfections for this complication and affect the review's results. In addition, the uncertainty and incompleteness in preparation methods, injection techniques, and filler dosages for various types of fillers make it difficult to reach definitive conclusions on the pathogenesis of FBGs. Being limited to the case size and the level of current evidence, the ability to provide clear recommendations on specific measures to reduce or manage this complication remains inadequate. This study aims to investigate the FBGs associated with collagen‐stimulating fillers using the PICO system for review. The results cannot serve as a standalone basis for determining the incidence of these fillers.

## 4 Conclusions

The tolerance and long‐term safety of microsphere‐based fillers for cosmetic indications still require close attention, despite being considered highly biocompatible materials with human tissue. This systematic review highlights the serious, albeit rare, potential complications of collagen stimulators and associated mechanisms and treatment recommendations for FBGs to further guide practitioners. To date, there is an absence of guiding consensus on the injection protocols and no complete effective method for treating FBGs, so prevention is the best solution to avoid unfavorable aesthetic outcomes, as with the granulomatous reaction of any other dermal fillers.

## Author Contributions

**He Qiu:** conceptualization, data curation, formal analysis, investigation, writing – original draft. **Hang Wang:** conceptualization, project administration, data curation, formal analysis. **Dongmei Wu:** methodology, formal analysis, investigation, supervision. **Chihchieh Lo:** methodology, formal analysis. **Shuo Liu:** methodology, formal analysis. **Qiang Ji:** formal analysis, investigation, supervision. **Al‐Attab Reem:** methodology, formal analysis.

## Ethics Statement

The authors have nothing to report.

## Consent

The authors have nothing to report.

## Conflicts of Interest

The authors declare no conflicts of interest.

## Supporting information


**Appendix S1:** Supporting Information.

**Table S1:** Supporting Information.

**Table S2:** Supporting Information.

## Data Availability

The data that support the findings of this study are available from the corresponding author upon reasonable request.

## References

[1] J. Ortiz‐Alvarez , J. A. Lebron‐Martin , L. Rodriguez Fernandez‐Freire , T. Zulueta Dorado , and J. S. Garcia Morillo , “Cutaneous and Ganglion Sarcoidosis Induced by Polycaprolactone Facial Filler: A New Expression of ASIA Syndrome?,” European Journal of Case Reports in Internal Medicine 8, no. 7 (2021): 002652, 10.12890/2021_002652. 34268272 PMC8276932

[2] S.‐L. Lin and M.‐O. Christen , “Polycaprolactone‐Based Dermal Filler Complications: A Retrospective Study of 1111 Treatments,” Journal of Cosmetic Dermatology 19, no. 8 (2020): 1907–1914, 10.1111/jocd.13518. 32485052 PMC7497126

[3] M. Ianhez , G. de Goés e Silva Freire , R. M. S. Sigrist , et al., “Complications of Collagen Biostimulators in Brazil: Description of Products, Treatments, and Evolution of 55 Cases,” Journal of Cosmetic Dermatology 23 (2024): 2829–2835, 10.1111/jocd.16343. 38693639

[4] F. Duranti , G. Salti , B. Bovani , M. Calandra , and M. L. Rosati , “Injectable Hyaluronic Acid Gel for Soft Tissue Augmentation – A Clinical and Histological Study,” Dermatologic Surgery 24, no. 12 (1998): 1317–1325, 10.1111/j.1524-4725.1998.tb00007.x. 9865196

[5] G. Lemperle , N. Gauthier‐Hazan , M. Wolters , M. Eisemann‐Klein , U. Zimmermann , and D. M. Duffy , “Foreign Body Granulomas After all Injectable Dermal Fillers: Part 1. Possible Causes,” Plastic and Reconstructive Surgery 123, no. 6 (2009): 1842–1863, 10.1097/PRS.0b013e31818236d7. 19483587

[6] J. M. C. Bohorquez and S. Desyatnikova , “Ultrasound‐Guided Treatment of Polycaprolactone Granuloma,” Plastic and Reconstructive Surgery 12, no. 2 (2024): e5610, 10.1097/gox.0000000000005610. 38348458 PMC10860983

[7] G.‐W. Hong , H. Hu , K. Chang , et al., “Review of the Adverse Effects Associated With Dermal Filler Treatments: Part I Nodules, Granuloma, and Migration,” Diagnostics 14, no. 15 (2024): 1640, 10.3390/diagnostics14151640. 39125515 PMC11311355

[8] W. Lee , S. Shah‐Desai , N.‐K. Rho , and J. Cho , “Etiology of Delayed Inflammatory Reaction Induced by Hyaluronic Acid Filler,” Archives of Plastic Surgery 51, no. 01 (2024): 20–26, 10.1055/a-2184-6554. 38425859 PMC10901605

[9] S. Liu and F. Xiong , “Injection‐Type Filler Useful in Stimulating Collagen Regeneration Remodeling in e.g Medical Cosmetology, Plastic Surgery, and Filling Comprises Filler Comprising Microsphere, Fiber and Matrix Material Preferably Hyaluronic Acid, Where Material of Microsphere and Fiber Comprises Polycaprolactone,” Patent CN117797314‐A.

[10] B. Quach and R. A. Clevens , “Complications of Injectables,” Atlas of the Oral and Maxillofacial Surgery Clinics 32, no. 1 (2024): 57–63, 10.1016/j.cxom.2023.10.005. 38307636

[11] H. Galadari , G. Krompouzos , M. Kassir , et al., “Complication of Soft Tissue Fillers: Prevention and Management Review,” Journal of Drugs in Dermatology 19, no. 9 (2020): 829–832, 10.36849/jdd.2020.5084. 33026743

[12] J. Woodward , T. Khan , and J. Martin , “Facial Filler Complications,” Facial Plastic Surgery Clinics of North America 23, no. 4 (2015): 447–458, 10.1016/j.fsc.2015.07.006. 26505541

[13] M. H. Gold and N. S. Sadick , “Optimizing Outcomes With Polymethylmethacrylate Fillers,” Journal of Cosmetic Dermatology 17, no. 3 (2018): 298–304, 10.1111/jocd.12539. 29602240

[14] C. Schardt , M. B. Adams , T. Owens , S. Keitz , and P. Fontelo , “Utilization of the PICO Framework to Improve Searching PubMed for Clinical Questions,” BMC Medical Informatics and Decision Making 7 (2007): 16, 10.1186/1472-6947-7-16. 17573961 PMC1904193

[15] S. Shahrabi‐Farahani , M. A. Lerman , V. Noonan , S. Kabani , and S. B. Woo , “Granulomatous Foreign Body Reaction to Dermal Cosmetic Fillers With Intraoral Migration,” Oral Surgery, Oral Medicine, Oral Pathology, Oral Radiology 117, no. 1 (2014): 105–110, 10.1016/j.oooo.2013.10.008. 24332334

[16] C.‐H. Lin , C.‐P. Chiang , B.‐Y. Wu , and H.‐W. Gao , “Filler Migration to the Forehead due to Multiple Filler Injections in a Patient Addicted to Cosmetic Fillers,” Journal of Cosmetic and Laser Therapy 19, no. 2 (2017): 124–126, 10.1080/14764172.2016.1248441. 27813656

[17] R. Poveda , J. V. Bagan , J. Murillo , and Y. Jimenez , “Granulomatous Facial Reaction to Injected Cosmetic Fillers – A Presentation of Five Cases,” Medicina Oral, Patología Oral y Cirugía Bucal 11, no. 1 (2006): E1–E5. 16388285

[18] M. Apikian , S. Roberts , and G. J. Goodman , “Adverse Reactions to Polylactic Acid Injections in the Periorbital Area,” Journal of Cosmetic Dermatology 6, no. 2 (2007): 95–101, 10.1111/j.1473-2165.2007.00303.x. 17524125

[19] Z. Saylan , “Facial Fillers and Their Complications,” Aesthetic Surgery Journal 23, no. 3 (2003): 221–224, 10.1067/maj.2003.45. 19336082

[20] A. Dionyssopoulos , A. Nikolis , A. Patsatsi , and D. Sotiriadis , “Granulomas of the Lips: A Rare Complication After Injection of Polylactic Acid for Aesthetic Augmentation,” Journal of Plastic, Reconstructive & Aesthetic Surgery 60, no. 9 (2007): 1079–1080, 10.1016/j.bjps.2007.04.002. 17532279

[21] O. Goldan , G. Garbov‐Nardini , E. Regev , A. Orenstein , and E. Winkler , “Late‐Onset Infections and Granuloma Formation After Facial Polylactic Acid (New‐Fill) Injections in Women who are Heavy Smokers,” Plastic and Reconstructive Surgery 121, no. 5 (2008): 336e–338e, 10.1097/PRS.0b013e31816b1234. 18453949

[22] S. Rastogi , L. L. Wang , C. Berry , et al., “Ruby Red Spheres in the Dermis: A Novel Histopathologic Finding of Poly‐L‐Lactic Acid Filler With Fite Staining,” Journal of Cutaneous Pathology 49, no. 10 (2022): 895–897, 10.1111/cup.14292. 35831239

[23] E. Skrzypek , B. Górnicka , D. M. Skrzypek , and M. R. Krzysztof , “Granuloma as a Complication of Polycaprolactone‐Based Dermal Filler Injection: Ultrasound and Histopathology Studies,” Journal of Cosmetic and Laser Therapy 21, no. 2 (2019): 65–68, 10.1080/14764172.2018.1461229. 29757038

[24] L. Or , J. A. Eviatar , G. G. Massry , F. P. Bernardini , and M. E. Hartstein , “Xanthelasma‐Like Reaction to Filler Injection,” Ophthalmic Plastic and Reconstructive Surgery 33, no. 4 (2017): 244–247, 10.1097/iop.0000000000000722. 27218809

[25] F. Philibert , V. Gras‐Champel , G. Chaby , et al., “Eruptive Granuloma After Injection of Ellansé® Successfully Treated Using Methotrexate,” Annales de Dermatologie et de Vénéréologie 147, no. 8–9 (2020): 525–529, 10.1016/j.annder.2020.02.009. 32276735

[26] S. Y. Moon , D. H. Eun , J. H. Park , et al., “Foreign Body Reaction Three Years After Injection With Polycaprolactone (Ellanse),” European Journal of Dermatology 27, no. 5 (2017): 549–551, 10.1684/ejd.2017.3089. 29084645

[27] C. M. Rudolph , H. P. Soyer , S. Schuller‐Petrovic , and H. Kerl , “Foreign Body Granulomas due to Injectable Aesthetic Microimplants,” American Journal of Surgical Pathology 23, no. 1 (1999): 113–117, 10.1097/00000478-199901000-00013. 9888711

[28] C. Requena , M. J. Izquierdo , M. Navarro , et al., “Adverse Reactions to Injectable Aesthetic Microimplants,” American Journal of Dermatopathology 23, no. 3 (2001): 197–202, 10.1097/00000372-200106000-00006. 11391099

[29] E.‐M. Reisberger , M. Landthaler , L. Wiest , J. Schroder , and W. Stolz , “Foreign Body Granulomas Caused by Polymethylmethacrylate Microspheres: Successful Treatment With Allopurinol,” Archives of Dermatology 139, no. 1 (2003): 17–20, 10.1001/archderm.139.1.17. 12533157

[30] K.‐J. Kim , H.‐W. Lee , M.‐W. Lee , J.‐H. Choi , K.‐C. Moon , and J.‐K. Koh , “Artecoll Granuloma: A Rare Adverse Reaction Induced by Microimplant in the Treatment of Neck Wrinkles,” Dermatologic Surgery 30, no. 4 Pt 1 (2004): 545–547, 10.1111/j.1524-4725.2004.30174.x. 15056148

[31] M. M. Al‐Qattan , “Late Artecoll Granulomas Aggravated by Pregnancy,” Annals of Plastic Surgery 58, no. 5 (2007): 592, 10.1097/01.sap.0000258127.15139.68. 17452859

[32] J. Fischer , G. Metzler , and M. Schaller , “Cosmetic Permanent Fillers for Soft Tissue Augmentation: A New Contraindication for Interferon Therapies,” Archives of Dermatology 143, no. 4 (2007): 507–510, 10.1001/archderm.143.4.507. 17438184

[33] A. Gelfer , A. Carruthers , J. Carruthers , F. Jang , and S. C. Bernstein , “The Natural History of Polymethylmethacrylate Microspheres Granulomas,” Dermatologic Surgery 33, no. 5 (2007): 614–620, 10.1111/j.1524-4725.2007.33123.x. 17451587

[34] A. G. Salles , P. H. Lotierzo , R. Gemperli , et al., “Complications After Polymethylmethacrylate Injections: Report of 32 Cases,” Plastic and Reconstructive Surgery 121, no. 5 (2008): 1811–1820, 10.1097/PRS.0b013e31816b1385. 18454007

[35] M. C. da Costa Miguel , C. F. W. Nonaka , J. N. dos Santos , A. R. Germano , and L. B. de Souza , “Oral Foreign Body Granuloma: Unusual Presentation of a Rare Adverse Reaction to Permanent Injectable Cosmetic Filler,” International Journal of Oral and Maxillofacial Surgery 38, no. 4 (2009): 385–387, 10.1016/j.ijom.2009.01.013. 19243914

[36] K. P. Santana , L. H. Pereira , O. Sabatovich , and A. Sterodimas , “Foreign‐Body Granulomas Caused by Polymethylmethacrylate (PMMA) Microspheres: Successful Correction by Autologous Fat Transplantation,” Journal of Plastic, Reconstructive & Aesthetic Surgery 63, no. 2 (2010): e139–e141, 10.1016/j.bjps.2009.01.088. 19345170

[37] M. M. Al‐Qattan , “Complications Related to Artecoll Injections for Soft Tissue Augmentation of the Hand: 3 Case Reports,” Journal of Hand Surgery 36, no. 6 (2011): 994–997, 10.1016/j.jhsa.2011.03.016. 21549527

[38] A. Lopez‐Pestana , A. Tuneu , C. Lobo , and J. Zubizarreta , “Sarcoid Granulomas in Facial Cosmetic Filler Material: Induction by Interferon‐ Alpha and Ribavirin in a Patient With Hepatitis C,” Actas Dermo‐Sifiliográficas 102, no. 9 (2011): 746–747, 10.1016/j.ad.2011.01.013. 21640959

[39] M. R.d. S. Quirino , A. C. C. Neves , M. S. Campos , A. A. H. Brandao , and A. L. Anbinder , “Oral Granuloma Formation After Injection of Cosmetic Filler,” Journal of Cranio‐Maxillofacial Surgery 40, no. 7 (2012): e194–e197, 10.1016/j.jcms.2011.10.007. 22100454

[40] W. Wu , P. Chayavichitsilp , and T. Hata , “Extremely Delayed Granulomatous Reaction to Soft‐Tissue Injectables (Polymethyl Methacrylate),” Journal of the American Academy of Dermatology 67, no. 5 (2012): e206–e207, 10.1016/j.jaad.2012.02.023. 23062915

[41] D. P. Friedmann , A. Kurian , and R. E. Fitzpatrick , “Delayed Granulomatous Reactions to Facial Cosmetic Injections of Polymethylmethacrylate Microspheres and Liquid Injectable Silicone: A Case Series,” Journal of Cosmetic and Laser Therapy 18, no. 3 (2016): 170–173, 10.3109/14764172.2015.1114642. 26735450

[42] A. Goldman and U. Wollina , “Polymethylmethacrylate‐Induced Nodules of the Lips: Clinical Presentation and Management by Intralesional Neodymium:YAG Laser Therapy,” Dermatologic Therapy 32, no. 1 (2019): e12755, 10.1111/dth.12755. 30285309

[43] M. Pompeu , V. Lima , H. L. Magalhaes , N. Regattieri , and I. M. C. Costa , “Foreign Body Granuloma Treatment With 22‐MHz Ultrasound‐Guided Corticoid Infiltration,” Journal of Cosmetic Dermatology 18, no. 3 (2019): 908–909, 10.1111/jocd.12750. 30160077

[44] S. M. Scofield‐Kaplan , S. Y. Patel , A. Mueller , et al., “Foreign‐Body Granulomata Caused by Injected Permanent Filler Masquerading as Cutaneous Sarcoidosis,” Ophthalmic Plastic and Reconstructive Surgery 35, no. 3 (2019): e82–e84, 10.1097/IOP.0000000000001385. 30921058

[45] A. D. McCarthy , S. Berkowitz , and W. G. Chernoff , “Successful Treatment of Noninflammatory CaHA Nodules Using Focused Mechanical Vibration Aesthetic Surgery Journal Open,” Forum 6 (2024): ojae018, 10.1093/asjof/ojae018. PMC11033680 38650973

[46] T. Daley , D. D. Damm , J. A. Haden , and M. T. Kolodychak , “Oral Lesions Associated With Injected Hydroxyapatite Cosmetic Filler,” Oral Surgery, Oral Medicine, Oral Pathology, Oral Radiology 114, no. 1 (2012): 107–111, 10.1016/j.oooo.2012.03.012. 22727099

[47] V. Sankar and H. S. McGuff , “Foreign Body Reaction to Calcium Hydroxylapatite After Lip Augmentation,” Journal of the American Dental Association 138, no. 8 (2007): 1093–1096, 10.14219/jada.archive.2007.0321. 17670876

[48] I. Moulonguet , E. Arnaud , P. Bui , and F. Plantier , “Foreign Body Reaction to Radiesse: 2 Cases,” American Journal of Dermatopathology 35, no. 3 (2013): E37–E40, 10.1097/DAD.0b013e3182732324. 23475146

[49] I. M. Vrcek , P. Malouf , and G. D. Gilliland , “A Novel Solution for Superficially Placed Calcium Hydroxylapatite (Radiesse) in the Inferior Eyelid,” Orbit (Amsterdam, Netherlands) 31, no. 6 (2012): 431–432, 10.3109/01676830.2012.694557. 22731869

[50] R.‐F. Liu , T.‐T. Kuo , Y.‐Y. Chao , and Y.‐H. Huang , “Alopecia With Foreign Body Granulomas Induced by Radiesse Injection: A Case Report,” Journal of Cosmetic and Laser Therapy 20, no. 7–8 (2018): 462–464, 10.1080/14764172.2018.1427867. 29482401

[51] S. Y. Huh , S. Cho , K. H. Kim , et al., “A Case of Complication After Matridex Injection,” Annals of Dermatology 22, no. 1 (2010): 81–84, 10.5021/ad.2010.22.1.81. 20548890 PMC2883406

[52] C. Massone , M. Horn , H. Kerl , C. M. Ambros‐Rudolph , A. M. G. Brunasso , and L. Cerroni , “Foreign Body Granuloma due to MatridexInjection for Cosmetic Purposes,” American Journal of Dermatopathology 31, no. 2 (2009): 197–199, 10.1097/DAD.0b013e318194816d. 19318810

[53] J.‐H. Yang , S.‐M. Lee , C.‐H. Won , et al., “Foreign Body Granuloma Caused by Hyaluronic Acid/Dextranomer Microsphere Filler Injection,” International Journal of Dermatology 51, no. 12 (2012): 1517–1518, 10.1111/j.1365-4632.2010.04795.x. 23171022

[54] G. Lemperle , V. Morhenn , and U. Charrier , “Human Histology and Persistence of Various Injectable Filler Substances for Soft Tissue Augmentation,” Aesthetic Plastic Surgery 27, no. 5 (2003): 354–366, 10.1007/s00266-003-3022-1. 14648064

[55] W. P. Werschler , “Oral Lesions Associated With Injected Hydroxyapatite Cosmetic Filler,” Oral Surgery, Oral Medicine, Oral Pathology, Oral Radiology 115, no. 3 (2013): 417–419, 10.1016/j.oooo.2012.10.025. 23380481

[56] T. Bhojani‐Lynch , “Late‐Onset Inflammatory Response to Hyaluronic Acid Dermal Fillers,” Plastic and Reconstructive Surgery 5, no. 12 (2017): e1532, 10.1097/gox.0000000000001532. 29632758 PMC5889432

[57] H. Kim , S. H. Cho , J. D. Lee , and H. S. Kim , “Delayed Onset Filler Complication: Two Case Reports and Literature Review,” Dermatologic Therapy 30, no. 5 (2017): e12513, 10.1111/dth.12513. 28585321

[58] O. Ibrahim , J. Overman , K. A. Arndt , and J. S. Dover , “Filler Nodules: Inflammatory or Infectious? A Review of Biofilms and Their Implications on Clinical Practice,” Dermatologic Surgery 44, no. 1 (2018): 53–60, 10.1097/dss.0000000000001202. 28538034

[59] A. B. Sadashivaiah and V. Mysore , “Biofilms: Their Role in Dermal Fillers,” Journal of Cutaneous and Aesthetic Surgery 3, no. 1 (2010): 2022, 10.4103/0974-2077.63257. PMC2890130 20606988

[60] R. J. Rohrich , G. Monheit , A. T. Nguyen , S. A. Brown , and S. Fagien , “Soft‐Tissue Filler Complications: The Important Role of Biofilms,” Plastic and Reconstructive Surgery 125, no. 4 (2010): 1250–1256, 10.1097/PRS.0b013e3181cb4620. 19935452

[61] O. Ciofu , C. Moser , P. O. Jensen , and N. Hoiby , “Tolerance and Resistance of Microbial Biofilms,” Nature Reviews. Microbiology 20, no. 10 (2022): 621–635, 10.1038/s41579-022-00682-4. 35115704

[62] C. Uruen , G. Chopo‐Escuin , J. Tommassen , R. C. Mainar‐Jaime , and J. Arenas , “Biofilms as Promoters of Bacterial Antibiotic Resistance and Tolerance,” Antibiotics‐Basel 10, no. 1 (2021): 3, 10.3390/antibiotics10010003. PMC7822488 33374551

[63] J. L. Balcazar , J. Subirats , and C. M. Borrego , “The Role of Biofilms as Environmental Reservoirs of Antibiotic Resistance,” Frontiers in Microbiology 6 (2015): 1216, 10.3389/fmicb.2015.01216. 26583011 PMC4628128

[64] D. Wolfram , A. Tzankov , and H. Piza‐Katzer , “Surgery for Foreign Body Reactions due to Injectable Fillers,” Dermatology 213, no. 4 (2006): 300–304, 10.1159/000096193. 17135735

[65] J. Emer and H. Sundaram , “Aesthetic Applications of Calcium Hydroxylapatite Volumizing Filler: An Evidence‐Based Review and Discussion of Current Concepts: Part 1 of 2,” Journal of Drugs in Dermatology 12, no. 12 (2013): 1345–1354. 24301235

[66] J. van Loghem , D. Funt , T. Pavicic , et al., “Managing Intravascular Complications Following Treatment With Calcium Hydroxylapatite: An Expert Consensus,” Journal of Cosmetic Dermatology 19, no. 11 (2020): 2845–2858, 10.1111/jocd.13353. 32185876 PMC7687073

[67] A. L. Lindgren and K. M. Welsh , “Management of Vascular Complications Following Calcium Hydroxylapatite Filler Injections: A Systemic Review of Cases and Experimental Studies,” Plastic and Aesthetic Research 9 (2022): 50, 10.20517/2347-9264.2022.09.

[68] M. Yankova , T. Pavicic , K. Frank , et al., “Intraarterial Degradation of Calcium Hydroxylapatite Using Sodium Thiosulfate – An In Vitro and Cadaveric Study,” Aesthetic Surgery Journal 41, no. 5 (2021): NP226–NP236, 10.1093/asj/sjaa350. 33544840

[69] P. del Barrio‐Diaz , C. Moll‐Manzur , S. Alvarez‐Veliz , and C. Vera‐Kellet , “Topical Sodium Metabisulfite for the Treatment of Calcinosis Cutis: A Promising New Therapy,” British Journal of Dermatology 175, no. 3 (2016): 608–611, 10.1111/bjd.14412. 26799451

[70] T. H. Park , “Usefulness of Surgical Approach for Granuloma Caused by CaHA Fillers,” Orbit 32, no. 1 (2013): 86, 10.3109/01676830.2012.737402. 23273043

