# Supplementary material for: Foreign Body Granulomas Reaction Related to Collagen Stimulatory Cosmetic Fillers: A Systematic Review
Source: J Cosmet Dermatol. 2025 Oct 24;24(10):e70459. doi: 10.1111/jocd.70459 (PMC12550546; doi:10.1111/jocd.70459)
Supplement: Supplementary file 1 — Appendix S1: Supporting Information. [file JOCD-24-e70459-s002.docx]

**Supplementary Information**

**Foreign Body Granulomas Reaction Related to Collagen Stimulatory Cosmetic Fillers: A Systematic Review**

**Search Strategies**

We employed the following key words and/or corresponding MeSH terms, combined with Boolean operators, to search for relevant literature in predefined electronic databases. The search strategy was as follows:

#1= (“complications” OR “adverse reactions”OR“lumps” OR “nodules” OR “granuloma ” OR “foreign body granuloma” OR “granulomatous foreign body reactions” OR “giant cell foreign reactions” OR “granulomatous reaction” OR “ lipogranuloma ”OR “foreign bodies” OR “granuloma, foreign body” OR “giant cells, foreign body” )

#2 = (“collagen biostimulators” OR “collagen stimulants” OR “stimulatory filler”)

#3 = (“Poly-L-lactic acid” OR “PDLLA” OR “PLLA” OR “Sculptra” OR “NewFill” OR “Elleva” OR “Löviselle” OR "CureWhite " OR “Lanluma V” OR “AestheFill” OR “Juvelook” OR “Lenisna”)

#4= (“polycaprolactone” OR “PCL” OR “ELLANSÉ”)

#5= (“ Dextran ” OR “Glucan” OR “Reviderm intra” OR “Matridex”)

#6= (“ calcium hydroxyapatite” OR “CaHA” OR “Radiesse”)

#7= (“ polyvinyl alcohol” OR “Bonita” OR “PVA” OR “PVOH”)

#8= (“polymethyl methacrylate” OR “PMMA” OR “Artecoll” OR “ArteFill” OR “Newplastic” OR “MetaCrill” OR “Lipen-10” OR “Arteplast” OR “Bellafill”)

#9= #2 OR #3 OR #4 OR #4 OR #5 OR #6 OR #7 OR #8

#10= #1 AND #7

**Eligibility criteria**

Referring to the internationally recognized PICO principles, we have clarified the inclusion criteria for cases as follows:

(1) P (Population): All patients included in the studies were plagued by cosmetic concerns and sought rejuvenation through aesthetic treatments using collagen-stimulating fillers.

(2) I (Intervention): Injection of collagen stimulators, either alone or in combination. The fillers involved are listed in Table 1.

(3) C (Control): The outcomes of all included cases were compared with their pre-injection status (without any signs of complications).

(4) O (Outcomes): FBGs or FBGs-induced lesions related to fillers after injection, such as nodules, lumps, swelling, or redness.

Since some cases exhibiting granulomatous reactions did not provide definitive histological information, a clinical diagnosis could still be made based on clinical history, presentation and other diagnostics supportives

. Therefore, after careful evaluation by all reviewers, these studies can be considered for inclusion in this study. The exclusion criteria relevant to the selection and data extraction are presented in Figure 1.
