# Supplementary material for: Foreign Body Granulomas Reaction Related to Collagen Stimulatory Cosmetic Fillers: A Systematic Review
Source: J Cosmet Dermatol. 2025 Oct 24;24(10):e70459. doi: 10.1111/jocd.70459 (PMC12550546; doi:10.1111/jocd.70459)
Supplement: Supplementary file 2 — Table S1: Supporting Information. [file JOCD-24-e70459-s001.docx]

**Supplemental Table 1. Common microsphere-based collagen stimulatory cosmetic fillers and its main feature**

| **Microsphere filling agent** | | **Commercial name** | **Main Carrier** | **Nature of microsphere substance** | **Histological feature of FBGs** |
| --- | --- | --- | --- | --- | --- |
| **Biodegradable microsphere filling agent** | PLLA-  based filler | Newfill | CMC and mannitol lyophilized powder | 10 to 125 μm in diameter | Histiocytes,  epithelioid cells, multinucleated giant cells, birefringence particles of different  size and shape and may contain asteroid bodies |
|  |  | Sculptra | CMC and mannitol lyophilized powder | Smooth surface and 40~63μm in diameter |  |
|  |  | Elleva | CMC and mannitol lyophilized powder | Smooth surface, and 40~63μm in diameter |  |
|  |  | Löviselle | CMC and mannitol lyophilized powder | Smooth surface, and 20~50 μm in diameter |  |
|  |  | Lanluma V | CMC and mannitol lyophilized powder | Not mention |  |
|  |  | CureWhite | HA gel | Smooth surfarce, and 20~45 μm in diameter |  |
|  | PDLLA-based filler | AestheFill | CMC gel | Smooth surface, and 30~70 μm in diameter |  |
|  |  | Juvelook | HA gel | Smooth surface, and 20~40 μm in diameter |  |
|  |  | Lenisna | HA gel | Smooth surface, and 40~80 μm in diameter |  |
|  | PCL-based filler | ELLANSÉ | CMC gel | Smooth surface, and 25~50 μm in diameter | Multiple small round cystic  spaces containing translucent  nonbirefringent microspheres  of approximately same size |
|  | CaHA-based  filler | Radiesse/Radiance | CMC and glycerin  gel | Smooth surface, and 25~45μm in diameter | Regularly sized, crackled bluish-gray spherules of calcium hydroxylapatite surrounded by  foreign body giant cells and epithelioid histiocytes |
|  | Dextran microspheres | Reviderm intra | HA gel | 40μm Sephadex | Multinucleated giant cells, lymphohistiocytic infiltration and bluish particles of dextran beads |
|  |  | Matridex | HA gel | Charged surface and 80~120 µm in diameter |  |
| **Non-biodegradable microsphere filling agent** | PMMA-based filler | Arteplast | Bovine collagen | Rough, irregular surfaces, a surface charge, and 20~40μm in diameter | Histiocytes, epithelioid cells, multinucleated giant cells, lymphocytes and mimicking fat cells made of microspheres of PMMA |
|  |  | Artecoll | Bovine collagen | Smooth surfaces, no electrical charge, and uniform size (30~42μm in diameter) |  |
|  |  | Artefill/Bellafill | Bovine collagen | Greater uniformity in microsphere size (30~50μm), surface contour, smoothness, and roundness |  |
|  |  | Newplastic | HA, D-1 propanediol, and pyrogenous solution | 30~103 μm in size and nonspherical, and particles conjoined |  |
|  |  | MetaCrill | Carboxygluconate | 1~80 μm in size |  |
|  |  | Lipen-10 | Cross-linked dextran, and hypromellose solution | 30~120 μm in size |  |
|  | PVOH-based filler | Bonita | Hydroxypropyl methyl cellulose  and HA | Smooth surface, and 25~45μm in diameter | Multinucle-ated giant cells, histiocytes, and lymphocyte around  cystic spaces containing  translucent, nonbirefringent  microspheres of  approximately same size |
|  |  | Evolution | Polyacrylamide gel | Irregular shape, poor surfaces and range from 1 to 80 μm in size |  |
