# Supplementary material for: Foreign Body Granulomas Reaction Related to Collagen Stimulatory Cosmetic Fillers: A Systematic Review
Source: J Cosmet Dermatol. 2025 Oct 24;24(10):e70459. doi: 10.1111/jocd.70459 (PMC12550546; doi:10.1111/jocd.70459)
Supplement: Supplementary file 3 — Table S2: Supporting Information. [file JOCD-24-e70459-s003.docx]

**Supplemental Table2. Basic characteristics of studies included for systematic review**

| Cosmetic procedures | References | Study types | Number of Cases  (M: F) | Mean age, ± SD (range) | Product for injection | Time until  complications | Presentation  symptoms | Treatment protocols | Outcome |
| --- | --- | --- | --- | --- | --- | --- | --- | --- | --- |
| PLLA | ^[15]^ | Case report | 12(F) | 56.83±10.84(range  from 44 to 78) | Sculptra | 5 to 36 months (median, 8 months) | Nodules in the maxillary, mandibular vestibular areas, the labial or buccal mucosa | Unknown | Unknown |
|  | ^[16]^ | Case report | 1(F) | 50 | Sculptra | 1 year | Forehead nodule | Lost follow up | Unknown |
|  | ^[17]^ | Case report | 1(F) | 55 | Unknown | 9.5 years | Elastic,  painless, tumefaction on the  enian, lip and  nasogenian sulcus areas | Oral deflazacort 15  mg per day for 10 days and then reducing the dosage  until withdrawing it completely 40 days | Tumefactions persisted on palpation |
|  | ^[18]^ | Case report | 4(F) | 52.50±4.65(range from 48 to 57) | 2.80±2.20 mL NewFill (range from 1to 6 mL) | 3.50±2.89 weeks (range from 1 to 6 weeks) | Multiple nodules in periorbital area | Surgical removal (Case one); Notreatment(Case two, three);Triamcinolone acetonide 10 mg/mL,Celestone, hronodose® and Kenacort A-10 (Case four) | Remission in symptoms |
|  | ^[19]^ | Case report | 12(F) | Unknown | New  fill | 2 months | Nodules followed with secondary infection | Not mentioned | Not mentioned |
|  | ^[20]^ | Case report | 1(F) | 45 | Unknown | 4 months | Numerous submucosal pale nodules on upper and lower lips | Triamcinolone acetonide injection three times at 6-week intervals | No completely resorbed |
|  | ^[21]^ | Case report | 4(F) | A mean age of 48 years | New  fill | Approximately 2 to 6 months | Pain, swelling, and erythema in lips | Steroid injection | No relief of symptoms |
|  | ^[22]^ | Case report | 1(M) | 55 | Sculptra | Over 10 years ago | Multiple subcutaneous non-tender nodules  on the arms, buttocks, and lower legs | Not mentioned | Not mentioned |
| PCL | ^[1]^ | Case report | 1(F) | 74 | Ellanse® | 3 months | Four subcutaneous and indurated nodules in both nasolabial folds and over zygomatic arches | Methotrexate (20 mg subcutaneous weekly) combination with prednisone (0.17 mg/kg/day) | Aesthetic and symptom improvement |
|  | ^[23]^ | Case report | 1(F) | 68 | 1 mL Ellansé-M | 1 year | Nodules accompanied by bluish skin discoloration developed within nasolabial fold | Surgical removal | Lost to follow up |
|  | ^[24]^ | A multicenter, retrospective study | 1(F) | 48 | Not mentioned | 6 months | Xanthelasma-like reaction in the lower eyelids | No treatment | Not mentioned |
|  | ^[25]^ | Case report | 1(F) | 47 | 2 mL Ellanse® | 9months | Multiple nodules on face | Oral methotrexate 10 mg per week for 3 months followed by 20 mg per week for 9 months | Complete regression |
|  | ^[26]^ | Case report | 1(M) | 36 | 2mL  Ellanse® | 3 years | Multiple asymptomatic firm, immobile subcutaneous nodules on both cheeks and both  infraorbital creases | Oral doxy  cycline (100 mg twice daily) for one month | Decreased in size. |
| PMMA | ^[27]^ | Case report | 3(F) | Not mentioned | Artecoll | Not  mentioned | Not  mentioned | Surgical removal | Not  mentioned |
|  | ^[28]^ | Case report | 1(M) | 52 | Artecoll | 6 months | Erythematous,  ribbed, palpable indurations of both nasolabial and fore-  head folds | Not  mentioned | Not  mentioned |
|  | ^[29]^ | Case report | 1(F) | 61 | 2.5mL Artecoll | 6 years | Red, firm nodules in forehead, tender to pressure | Allopurinol initiated at 200 mg/d and increased to a maximum of 600 mg/d after 4 weeks (Total of 24 weeks) | Symptom improvement |
|  | ^[30]^ | Case report | 1(F) | 50 | Artecoll | 4 years | Extensive nodules in the neck | Three intralesional injections of triamcinolone acetonide | Lesions disappeared |
|  | ^[31]^ | Case report | 1(F) | 30 | Artecoll | 3  years | Multiple lumps  in the face | Not receive any management | No follow-up |
|  | ^[32]^ | Case report | 1(F) | 48 | Artecoll | 10 years | Blue-red swelling in the upper and lower lips, nasolabial grooves, and glabella | Oral allopurinoland surgical removal | Remission in symptoms |
|  | ^[33]^ | Case report | 10 (1M:9F) | 47.1±9.02(range from 38 to 64) | Artecoll | 2 to 48 months (median, 23.7 months) | Multiple nodules  in the face | Intralesional injection of corticosteroids | 70% cases resolved within  2 years ;30%  cases no relief |
|  | ^[34]^ | Case report | 10(2M:8F) | 47.4±11.57(range from 32 to 70) | Artecoll;Bioplasty;Metacrill | 1 to 12 months (median, 4.3 months) | Multiple clinical nodules | Unknown | Unknown |
|  | ^[35]^ | Case report | 1(F) | 56 | Unknown | 1 year | Swelling and nodule in the lower lip | Surgical removal | Remisson |
|  | ^[36]^ | Case report | 1(F) | 59 | Unknown | 5 years | Face and neck granulomas | Autologous fat transplantation | Facial contouring improvement |
|  | ^[37]^ | Case report | 3（F） | 53.3±10.41(range from 45 to 65) | Artecoll | 1 to 3 years (median, 2 years) | Hand lumps | Local injections of  triamcinolone | Symptoms resolve and  lumps persisted |
|  | ^[38]^ | Case report | 1（F） | 56 | Artecoll | 14 years | Facial multiple nodules | Stopping antiviral treatment | Spontaneous regression of the lesions |
|  | ^[39]^ | Case report | 1（F） | 54 | Artecoll | 2 years | Multiple oral and perioral indurations | Intralesional injection of corticosteroids | Nodules disappeared or reduced in size |
|  | ^[40]^ | Case report | 1M | 72 | Unknown | 15 years | Noduleson bilateral cheeks, lower lip, and chin | Intralesional corticosteroid injections | Decrease in size and palpability of nodules |
|  | ^[41]^ | Case report | 1(F) | 57 | Unknown | 3 years | Progressive, culture-  negative inflammatory glabellar lesion | Combination therapy with minocycline, 595-nm pulsed dye laser, intralesional 5-fluorouracil or triamcinolone | Significant improvement |
|  | ^[42]^ | Case report | 2(F) | 50 to 55 (median, 52.5) | Unknown | 2 to 6 years (median, 4 years) | Progressive nodules in the face and lips | Intralesional neodymium:YAG laser and suction | Resolution |
|  | ^[43]^ | Case report | 1(F) | 69 | Unknown | 9 years | Palpable, cold, painless, and immobile nodules in the bilateral nasolabial folds | Intralesional corticoid infiltration guided by 22-MHz ultrasound | Resolution |
|  | ^[44]^ | Case report | 1(F) | 75 | Artefill | Unknown | Swelling and subcutaneous nodules in the infraorbital area | Surgical removal | Resolution |
| CaHA | ^[15]^ | Case report | 13(F) | 56.31±10.69(rang from 35 to 71) | Radiesse | 2 to 12 months (median, 6 months) | Nodules in the initial sites (labial mucosa, skin of the chin, or nasolabial fold) and away from the injection sites (the skin of the nasolabial fold, commissure, or mental region) | Unknown | Unknown |
|  | ^[24]^ | A multicenter, retrospective study | 4(F) | 49.25±6.14(rang from 42 to 57) | Radiesse | 10 to 14 months (median, 12 months) | Swelling and yellow deposits of the lower eyelids | Steroid injection, fractionated laser (case one); CO_2_ laser (case two); Steroid injection, 5FU injection, direct excision (case three); No treatment (case four) | Subtotal im-  provement（case one）; Incomplete resolution (case two);Remisson (case three);Unknown(case four) |
|  | ^[45]^ | Case report | 1(F) | Not mentioned | Radiesse | 48 hours | Bilateral noninflammatory nodules in the jowls | Focused mechanical vibration | Resolved |
|  | ^[46]^ | Case report | 8(1M:7F) | 54.88±9.16 (range from 41 to 72) | Radiesse | Not mentioned | Lower lip, upper lip, or mandibular labial vestibule masse | Surgical removal | Not  mentioned |
|  | ^[47]^ | Case report | 1(F) | 51 | Radiesse | A few weeks | Lower lip nodule | Surgical removal | Remisson |
|  | ^[48]^ | Case report | 2(F) | 46 | Radiesse | About 11 months | Nose and cheek mass | Surgical removal | Resolved |
|  | ^[49]^ | Case report | 2(F) | 48.5 | Radiesse | One month | Erythema and induration of inferior periorbita | Saline injections and erbium laser treatments | Resolved |
|  | ^[50]^ | Case report | 1(F) | 44 | Radiesse | 1 month | Alopecia | Not receive any management | Remisson |
| Dextran microspheres | ^[51]^ | Case report | 1(F) | 56 | Matridex | 5 Weeks | Solitary indurated erythematous nodule in glabellar fold | Oral doxycycline and intralesional injections of triamcinolone acetonide | Partial improvement |
|  | ^[52]^ | Case report | 1(F) | 43 | Matridex | 4 Weeks | Multiple, painful, reddish, nonulcerated, hard nodules on both cheeks and periocular regions | Systemic antibiotic treatment with  Cephalexin and topical treatment with methylprednisolone aceonate | Resolution with residual post inflammatory hyperpigmentation |
|  | ^[53]^ | Case report | 1(F) | 58 | Matridex | 5  months | Round, slightly ten-  der, firm nodular lesions in the periorbital area | Intralesional injections of triam  cinolone | Mild  improvement |
